# Surveillance of Pediatric Invasive Bacterial Diseases in the Veneto Region: Epidemiological Trends and Outcomes over 17 Years (2007–2023)

**DOI:** 10.3390/vaccines13030230

**Published:** 2025-02-24

**Authors:** Silvia Cocchio, Claudia Cozzolino, Andrea Cozza, Patrizia Furlan, Enrica Frasson, Sara Tarantino, Elisabetta Conte, Lorenzo Chiusaroli, Irene Amoruso, Francesca Zanella, Davide Gentili, Michele Tonon, Francesca Russo, Tatjana Baldovin, Vincenzo Baldo

**Affiliations:** 1 Department of Cardiac, Thoracic, Vascular Sciences, and Public Health, University of Padua, 35128 Padua, Italy; silvia.cocchio@unipd.it (S.C.); claudia.cozzolino@phd.unipd.it (C.C.); andrea.cozza@studenti.unipd.it (A.C.); patrizia.furlan@unipd.it (P.F.); enrica.frasson@studenti.unipd.it (E.F.); sara.tarantino@studenti.unipd.it (S.T.); elisabetta.conte.2@studenti.unipd.it (E.C.); irene.amoruso@unipd.it (I.A.); tatjana.baldovin@unipd.it (T.B.); 2 Preventive Medicine and Risk Assessment Unit, Azienda Ospedale University of Padua, 35128 Padua, Italy; 3 Division of Pediatric Infectious Diseases, Department for Women’s and Children’s Health, University of Padua, 35128 Padua, Italy; lorenzo.chiusaroli@phd.unipd.it; 4 Regional Directorate of Prevention, Food Safety, Veterinary, Public Health—Veneto Region, 30123 Venice, Italy

**Keywords:** pediatric, meningitis, sepsis, surveillance system, *Neisseria meningitidis*, *Streptococcus pneumoniae*, *Haemophilus influenzae*, *Streptococcus agalactiae*, *Streptococcus pyogenes*, vaccination

## Abstract

**Introduction:** Invasive bacterial diseases (IBDs) such as meningitis and sepsis are significant public health concerns, particularly in pediatric populations. This study analyzes the incidence, outcomes, and bacterial serotype distribution of pediatric IBDs in the Veneto Region over 17 years. **Methods:** An observational study was conducted using data (2007–2023) from the surveillance system of the Veneto Region, including microbiologically confirmed cases in individuals < 18 years. Differences by age groups and trends were statistically assessed. **Results:** A total of 535 pediatric IBD cases were reported, with *Streptococcus pneumoniae* (54.6%), *Neisseria meningitidis* (19.6%), and *Streptococcus agalactiae* (13.5%) being the most common pathogens. *Haemophilus influenzae* infections were more commonly represented in infants under 1 year (41.5%), whereas *S. pneumoniae* and *N. meningitidis* were more frequent in the 1–4-year age group (40.8% and 37.1%, respectively). Sepsis was the most common clinical presentation (57.2%), followed by meningitis (36.3%). Serotype analysis revealed that *S. pneumoniae* serotype 3 was the most prevalent, while serogroup B dominated *N. meningitidis* cases. Temporal trends generally showed a decline in cases until 2019, a drop during the COVID-19 pandemic, and a subsequent resurgence in 2022–2023. **Conclusions:** Our research underscores the value of evidence-based epidemiology through robust surveillance systems in tracking IBD trends and serotype shifts, essential for guiding vaccination strategies and public health interventions. These insights highlight the effectiveness of vaccination programs and the necessity of ongoing monitoring to inform public health policies. Improved data integration and completeness are recommended to enhance surveillance accuracy.

## 1. Introduction

Invasive bacterial diseases (IBDs), including meningitis, bacteremic pneumonia, and other infections characterized by bacterial isolation from normally sterile sites, represent a cause of severe morbidity and mortality in pediatric populations. In particular, children under five years of age and those with underlying medical conditions, such as immunodeficiencies, prematurity, or chronic illnesses, are at the highest risk of developing IBDs.

Among pediatric individuals, the epidemiology of causative bacterial organisms is more heterogeneous than in adults, significantly varying by age [1]. The etiological agents most commonly associated with pediatric IBDs are *Neisseria meningitidis* (meningococcus), *Streptococcus pneumoniae* (pneumococcus), and *Haemophilus influenzae* [2,3]. Other microorganisms are recognized contributors (causing 90–95% of infections with the aforementioned bacteria): *Staphylococcus aureus*, *Streptococcus agalactiae*, *Streptococcus pyogenes*, *Listeria monocytogenes*, and *Salmonella* spp. [1].

Clinical presentation of these infections is often nonspecific, complicating early diagnosis and delaying appropriate treatment. Fever represents the main symptoms in children, making it challenging to determine the causative agent without targeted diagnostic tests [4].

Considering the specific etiological agents, *Streptococcus pneumoniae* is the leading cause of bacterial meningitis and community-acquired bacterial pneumonia; this bacterium can also cause osteomyelitis. The age groups most at risk are young children under 5 years and older adults [5]. *Neisseria meningitidis* is also responsible for meningitis and sepsis. Less common presentations of invasive meningococcal diseases (IMDs) include pneumonia (5% to 15% of cases), arthritis (2%), otitis media (1%), and epiglottitis (less than 1%) [6]. The age group most affected is children under 5 years of age, but adolescents and young adults up to 25 years old are also at increased risk. *Haemophilus influenzae* can instead cause localized respiratory infections (otitis, sinusitis, bronchitis) or invasive forms such as bacteremia, sepsis, pneumonia, meningitis, arthritis, pericarditis, facial cellulitis, and osteomyelitis. Invasive *H. influenzae* disease primarily affects newborns and children under 5 years of age, as well as elderly individuals over 65, especially those with comorbidities or immunosuppression.

It is estimated that healthy carriers in the general population range from 5% to 10% of healthy adults (20% to 40% of healthy children) for *S. pneumoniae*, from 5% to 10% for *N. meningitidis* (up to 30% in teenagers and young adults), and from 20% to 80% in children (increasing with age) for *H. influenzae* [7,8,9].

The latest surveillance reports from the European Centre for Disease Prevention and Control (ECDC) indicate that in 2018, there were 24,663 confirmed cases of invasive pneumococcal disease (IPD) in the European Union/European Economic Area (EU/EEA), with a notification rate of 14.4 cases per 100,000 population in infants under 1 year, and about 5.5 cases per 100,000 in children aged 1 to 4 years (all-ages rate was estimated at 6.4 cases per 100,000) [10]. Additionally, there were 1698 confirmed cases of invasive *Haemophilus influenzae* disease, with a notification rate of 4.0 cases per 100,000 population in infants under 1 year, and about 1 per 100,000 in children aged 1 to 4 years (all-ages rate was estimated at 0.4 cases per 100,000) [11,12]. In 2022, 1149 confirmed cases of IMD, including 110 deaths, were reported in 30 EU/EEA Member States. The notification rate for IMD rose to 0.3 cases per 100,000 population, following a decline in IMD incidence in 2020 and 2021, coinciding with the COVID-19 pandemic [13]. Age-specific rates were estimated at 4.3 per 100,000 population in infants under 1 year, and about 0.8 per 100,000 in children aged 1 to 4 years [13].

Regarding *Streptococcus agalactiae*, known as group B streptococcus (GBS), it remains one of the leading causative agents of invasive bacterial infections in infants (<1 year) and significantly contributes to neonatal mortality worldwide [14]. Group A streptococcus (GAS, or *S. pyogenes*) is also commonly associated with mild illnesses such as sore throat, headache, scarlet fever, and pharyngitis during winter months among kindergarten and school-aged children, and the ECDC has reported an alarming increase in invasive infections since September 2022 [15].

The majority of IBDs are vaccine-preventable diseases; in fact, currently, vaccines are available to protect against meningococcal serogroups A, B, C, Y, and W; up to 23 different pneumococcal serotypes; and *H. influenzae* type b (Hib).

Vaccination has played a key role in reducing the incidence of several bacterial infections in children. Indeed, the majority of IBDs are vaccine-preventable diseases and the introduction of conjugate vaccines, such as the pneumococcal conjugate vaccine (PCV), *Haemophilus influenzae* type b (Hib) vaccine, and meningococcal vaccines, has significantly decreased the prevalence of diseases caused by these pathogens [16,17]. The current vaccination schedule in the Veneto Region, approved in August 2023, follows the guidelines provided by the Italian National Vaccine Prevention Plan 2023–2025 [18] (see Table A1).

Further, the COVID-19 pandemic period has impacted the epidemiology of IBDs: indeed, while the total pediatric visits decreased due to reduced transmission of common viral infections, some studies have reported an increase in the number of serious bacterial infections (SBIs) and IBIs during the pandemic period [19]. However, the current epidemiological trends during the COVID-19 pandemic are still under investigation, and the pre- and post-incidence of IBDs is still unclear.

The purpose of this study is therefore to estimate the incidence of IBDs in the pediatric population of the Veneto Region based on regional surveillance data from 2007 to 2023, assessing evolutions in the distribution of associated etiological agent serogroups or serotypes.

## 2. Materials and Methods

### 2.1. Study Population and Data Source

This observational study analyzed notification reports of invasive bacterial diseases captured by the surveillance system of the Veneto Region in Italy. As of 2024, the Veneto Region had a population of 4.8 million, with an average age of 46.9 years, ranking 13th out of Italy’s 19 regions and two autonomous provinces in terms of population aging [20,21]. In detail, the Veneto Region is demographically structured as follows: 12.0% are aged 0–14 years, 63.5% fall within the 15–64 age range, and 24.5% are aged 65 years and older, with a dependency ratio of 57.5.

In 2023, the Veneto Region recorded a birth rate of 6.3 per 1000 inhabitants, with an average fertility rate of 1.21 children per woman and an average maternal age at childbirth of 32.7 years [20,21].

In terms of healthcare, the Veneto Region, like the rest of Italy, is served by the National Health Service (NHS). The NHS ensures universal coverage for the entire population and is funded through general taxation [22,23,24]. Healthcare services are coordinated regionally and delivered by local authorities.

The surveillance of invasive bacterial infections in the Veneto Region relies on three distinct data sources and has evolved over the years into a single computerized system for case recording, Sistema Informativo Regionale Malattie Infettive (SIRMI) [25], enabling comprehensive and unified management of all notifications, as previously described [22,26].

Regional surveillance encompasses all microorganisms capable of causing invasive diseases, especially *Streptococcus pneumoniae*, *Neisseria meningitidis*, and *Haemophilus influenzae* [22]. Mandatory notification forms include information on the place and date of notification, age, sex, sampling materials, clinical presentation, etiological agent and serotype, place and date of symptom onset, vaccination status, outcome (recovery, death), and sequelae (chronic condition following as a consequence of IBDs). However, there have been variations over the years, and not all fields are consistently completed.

### 2.2. Inclusion Criteria and Case Definition

Our study focused on all pediatric cases of IBD reported during the period 2007–2023 by local health authorities through the surveillance system portal of the Veneto Region and confirmed by microbiology laboratories specifically designated for such confirmation. The cohort was limited to individuals < 18 years. Notifications related to individuals not residing in the Veneto Region were excluded. As previously described [22,26], a case of invasive bacterial disease was defined as the isolation of a bacterial pathogen from blood or another normally sterile site, in line with the EU case definition [27,28]. Bacterial samples requiring confirmation were sent to the regional reference laboratory, where pathogen identification was carried out using standardized methods and procedures in accordance with protocol. When possible, pathogens were also serotyped [22,26].

### 2.3. Statistical Analysis

Frequencies and percentages were used to represent the variables. Pearson’s Chi-square test and, alternatively, Fisher’s exact test, were used to assess differences in the notification variables of invasive bacterial diseases across different age groups.

Analogously to Cocchio et al. [22], annual notification rates of IBD per 100,000 were calculated by dividing the number of confirmed cases by the estimated size of the resident pediatric population for each age group [20]. Rates were standardized by age using the direct method, with the 2015 Veneto Region population serving as the reference standard. The case fatality rate (CFR) was calculated by dividing the number of reported deaths by the number of notifications, expressed as a percentage.

Joinpoint regression [29] was performed to assess the significance of trends in the notification rate over the years of interest. Results were expressed as annual percentage change (APC) and average APC (AAPC).

Confidence intervals (CIs) were calculated as appropriate. A *p*-value < 0.05 was considered significant for results. Python 3.8.18 and R 4.2.2 software were used for data processing, analysis, and visualization.

### 2.4. Ethics Statement

IBD notification records were obtained from the administrative databases of the Veneto Region, and the disclosure and utilization of such records for educational and scientific purposes do not necessitate approval from ethical committees. On 24 January 2023, the Veneto Region implemented a code of conduct for the use of health data for educational and scientific publication purposes (Official Bulletin of the Region, “*Bollettino Ufficiale della Regione*” no. 10), as established by the European Committee (European Regulation 2016/679). This implementation previously received approval from the Italian Personal Data Protection Authority on 14 January 2021.

Adhering to the current Italian privacy legislation, the publication and utilization of health data, along with the processing methods, must occur exclusively in aggregate form, without any reference to patients’ personal information. Prior to providing access to the authors, all personal data that could potentially lead to identification were substituted with anonymous codes, in accordance with current privacy regulations (Legislative Decree no. 196 of 30 June 2003).

## 3. Results

### 3.1. Characterization of Pediatric IBD Cases

In total, 535 cases of pediatric invasive bacterial disease were reported to the regional surveillance system from 2007 to 2023. More than half of the subjects were infants (<1 year of age, 37.2%), or toddlers or preschoolers aged 1–4 years (34.0%). Older children aged 5–9, 10–14, and 15–17 years represented 17.0%, 5.4%, and 6.4% of the sample, respectively. There was a general male predominance (58.5% males versus 41.5% females). The IBD cases are described in Table 1.

The most prevalent etiological agent was *Streptococcus pneumoniae* (54.6%), followed by *Neisseria meningitidis* (19.6%) and *Streptococcus agalactiae* (13.5%).

Significant differences in the distribution of causative microorganisms were observed across age groups (*p* < 0.0001). *Streptococcus pneumoniae* was the leading cause of IBDs in all age groups except for adolescents aged 15 years and older, where *Neisseria meningitidis* was the dominant pathogen (58.8% of cases). Overall, the prevalence of *N. meningitidis* increased among individuals older than 10 years compared to younger children (31.0% in those aged 10–14 years versus 8.8% to 21.4% in those younger than 10 years). Notably, *S. agalactiae* was responsible for IBDs almost exclusively in infants, where it ranked second in occurrence (35.2%).

Regarding the age distribution of notifications by etiological agents, *S. agalactiae* and *H. influenzae* infections are more commonly represented in infants under 1 year (accounting for 97.2% and 41.5% of cases, respectively), whereas *S. pneumoniae*, *N. meningitidis*, and *S. pyogenes* are more frequent in the 1–4 years age group (40.8%, 37.1%, and 44.0%, respectively).

The majority of cases were microbiologically confirmed by isolating bacteria from blood (64.7%) and from cerebrospinal fluid (31.8%).

Associated meningitis, sepsis, bacteremic pneumonia, and other invasive diseases were reported in 36.3%, 57.2%, 15.1%, and 6.5% of notifications, respectively.

The frequency of associated diseases differed significantly across age groups. Sepsis was the most common invasive diagnosis in children aged 0–9 years (<1 year: 69.3%; 1–4 years: 49.5%; 5–9 years: 57.1%), while meningitis was predominant in children and adolescents older than 10 years (10–14 years: 44.8%; 15+ years: 61.8%).

About 41.2% and 45.1% of all reported meningitis and sepsis cases were observed in the age group under one year. Bacteremic pneumonia and other clinical presentations were more commonly reported in notifications concerning the 1–4 years age group (54.3% and 37.1%, respectively).

Specifically, meningitis was reported in 70.5% of *N. meningitidis* cases, rising to 80% among those aged >15 years.

In 80% and 76% of notifications for *S. agalactiae* and *S. pyogenes*, respectively, an associated sepsis was reported. In contrast, among notifications for *S. pneumoniae*, *N. meningitidis*, and *H. influenzae*, the percentages were 54.8%, 50.5%, and 41.5%, respectively.

Sequelae following an IBD were observed in five subjects (0.9%) and were evenly distributed among pathogens (except *S. pyogenes*, for which no cases were reported). A 3-year-old male toddler with IPD and a 12-year-old male with IMD developed audiological consequences, a newborn female with meningitis and sepsis caused by *S. agalactiae* developed neurological sequelae and thrombosis, a 5-year-old girl developed pansinusitis and bilateral acute otitis media following *H. influenzae* sinusitis and bacteremia, and another newborn female had other unspecified sequelae.

Among the pediatric IBD notifications reported through surveillance, we observed 24 cases with fatal outcomes, of whom 17 (70.8%) had an invasive presentation with sepsis. Over 41% of deaths occurred in the 1–4 years age group, while 25% were in infants under 1 year.

Survival information was missing for more than 360 cases, resulting in a case fatality rate (CFR) of 14.0% among cases with recorded outcomes (171, 32.0%).

Stratifying by age, the CFR was highest in the 10–14 years group (20.0%) and the 1–4 years group (18.2%), while it was 9.4%, 12.5%, and 15.4% in infants < 1 year, 5–9 years, and those over 15 years, respectively. When grouped by etiological agent, the CFR was, in descending order, 40.0% for *S. pyogenes*, 34.2% for *N. meningitidis*, 9.4% for *S. pneumoniae*, 4.8% for *H. influenzae*, and 0% for *S. agalactiae*.

### 3.2. Trends in Notification and Case Fatality Rate

**Figure 1:**
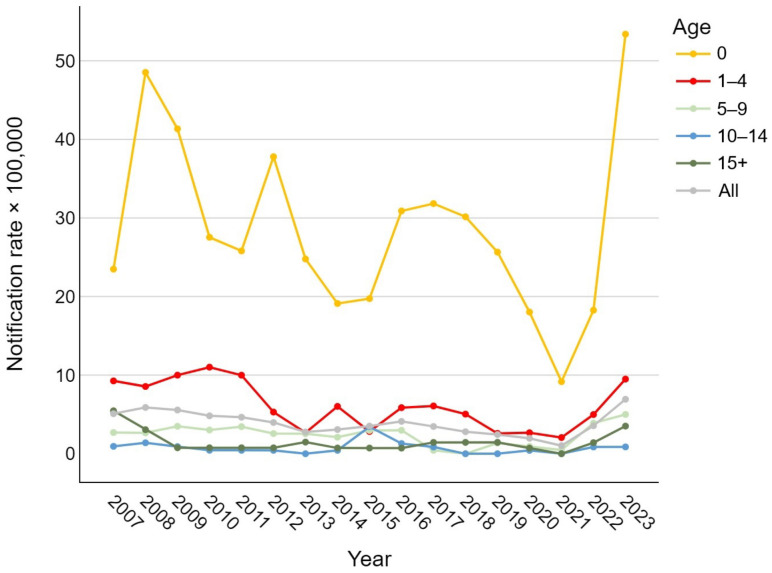
Age-specific trend of pediatric invasive bacterial disease notification rates per 100,000 in the Veneto Region from 2007 to 2023.

Analyzing the trend in notification rates over time, Figure 1 shows a general decrease in the overall number of IBD cases, regardless of the etiological agent, during the period from 2007 to 2019. During the SARS-CoV-2 pandemic, a sharp decline in notification rates was observed (1.0 per 100,000), followed by a steep rise in 2022 and 2023, reaching or exceeding pre-2010 values for nearly all age groups (overall 6.9 cases per 100,000). In newborns, the rate ranged from 9.2 to 53.4 cases per 100,000; in the 1–4 age group, between 2.1 and 11.0 per 100,000; in the 5–9 and over-15 groups, between 0 and about 5 per 100,000; and in the 10–14 group, between 0 and 3.4 per 100,000.

**Figure 2:**
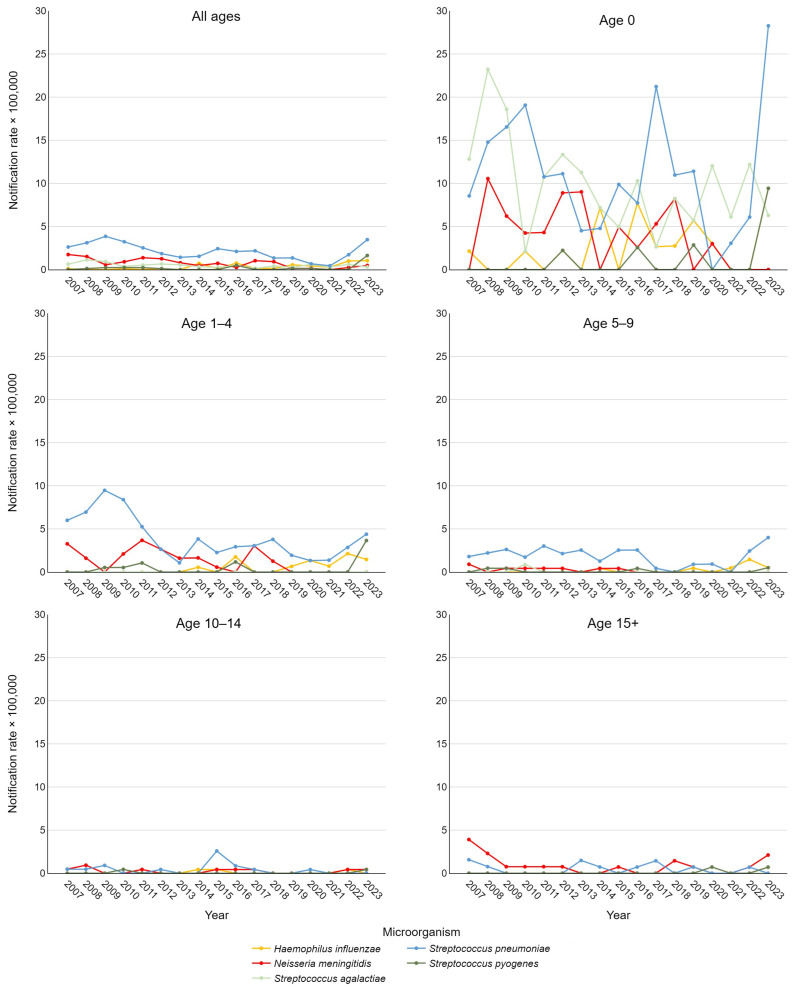
Trend of pediatric invasive bacterial disease notification rate per 100,000 in the Veneto Region from 2007 to 2023, stratified by etiological agent and age group.

Figure 2 depicts notification rates stratified by etiological agent and age group. Using Joinpoint analysis to examine these time trends, significant findings emerged. A decrease in IBDs caused by *S. pneumoniae* was observed in the 5–9 age group from 2007 to 2020 (APC = −7.78, 95% CI −32.21; −0.43) and across all pediatric cases (0–17 years) from 2018 to 2021 (APC = −32.6, 95% CI −48.92; −11.74). For the overall pediatric cohort, a significant positive trend in IPD was also noted between 2021 and 2023 (APC = 178.23, 95% CI 48.62; 336.02). For IMD, significant declines were observed in adolescents over 15 years from 2007 to 2009 (APC = −59.64, 95% CI −72.17; −13.46) and throughout the entire study period (AAPC = −7.88, 95% CI −12.16; −0.04). This result was consistent when considering all pediatric cases, with an AAPC of −10.91 (95% CI −17.47; −3.86).

For *S. pyogenes*, a statistically significant positive trend was observed across the entire population from 2019 to 2023 (APC = 71.1, 95% CI 2.22; 154.55). Additionally, a positive AAPC for the entire study period approached significance (*p* < 0.1), as did the AAPC for rates in newborns only. For *S. agalactiae* and *H. influenzae*, AAPCs approaching significance were found considering all ages, with a slightly negative trend for the former and a positive trend for the latter.

Given the high proportion of cases with missing survival data, it was not possible to analyze time trends in the CFR. Examining the absolute number of deaths, there were between one and three deaths per year from 2007 to 2016, between zero and one death per year from 2017 to 2022, and three deaths in 2023.

### 3.3. Evolution of Serotype Distribution

**Figure 3:**
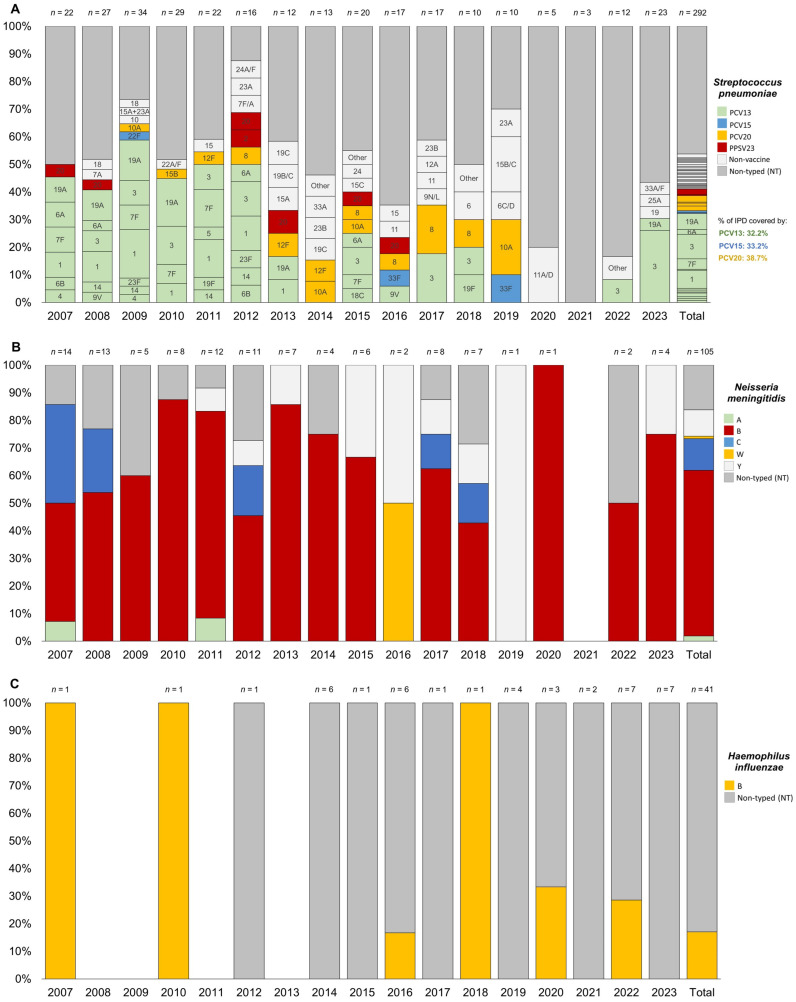
Trend of serotype distribution for pediatric (0–17 years) invasive bacterial disease caused by *Streptococcus pneumoniae* (**A**), *Neisseria meningitidis* (**B**), and *Haemophilus influenzea* (**C**) in the Veneto Region from 2007 to 2023.

The most frequent serotypes for *S. pneumoniae* were serotype 3 (8.9%), followed by serotype 1 (6.5%), serotype 19A (5.8%), serotype 7F (3.8%), and serotype 8 (2.4%). More than 12% of the isolated serotypes were non-vaccine (those not included in PCV20, previous PCV versions, or PPSV23). Notably, 46.2% of the total IPD cases were not typed or typeable (see Figure 3A).

For *N. meningitidis*, the most frequent serogroups were B (60.0%), C (11.4%), and Y (9.5%), with 16.2% of cases untyped or untypable (see Figure 3B).

For *H. influenzae*, all typed cases (only 17.1% of the total) were associated with serotype b (see Figure 3C).

For the other detected streptococci, further classification beyond the serogroup was not performed, only distinguishing between group A (*S. pyogenes*) and group B (*S. agalactiae*), without testing for serotypes.

Figure 3 showed a consistent presence over the years, representing up to 26% of the total cases per year (Figure 3A). Serotype 1 was no longer detected after 2014. Similarly, serotype 19A was absent after 2014 but reappeared with one new case of IPD in children aged 1–4 years in 2023. Serotypes 7F and 20 were observed only in children under 10 years and became undetectable after 2017. In contrast, serotype 8 appeared to emerge in 2012 and has since been circulating across all age groups. Among this pediatric population, non-vaccine serotypes increased significantly, nearly doubling after 2012 and reaching up to 40% in 2019.

Stratifying by age, the trends among infants under 1 year and children aged 1–4 years mirror those observed in the overall pediatric population under 18 years (see Supplementary Materials Figure S1).

For *N. meningitidis*, serogroup B was generally the predominant serogroup throughout the entire study period. However, when stratified by age see (Supplementary Materials Figure S2), it was particularly prominent in all years among children under 5 years old. In fact, most cases of serogroup C occurred in (pre)adolescents over 10 years of age in the 2007 and 2008 cohorts, while only sporadic cases were reported in children aged 1–4 years after 2012. Serogroup C was no longer detected after 2019.

Cases of IMD due to serogroup Y emerged only after 2011, affecting neonates and (pre)adolescents over 10 years old, with two cases in 2015 and 2016, and generally no more than one case annually through 2023. For serogroups A and W, single cases were reported in a child aged 5–9 years in 2007 and in a neonate in 2016, respectively.

For *H. influenzae*, there has been an increase in untyped cases since 2016, particularly in the 1–4 and 5–9 age groups, although the trends were not statistically significant in the Joinpoint analysis described previously (see Supplementary Materials Figure S3).

## 4. Discussion

This observational study estimated the incidence of IBDs in the pediatric population of the Veneto Region based on regional surveillance data from 2007 to 2023 and evaluated the evolution in the distribution of serotypes or serogroups of the associated etiological agents. Our analysis revealed that children younger than one year constituted the most affected age group, consistent with the latest surveillance data from the ECDC [10,12,13]. This finding may be attributed to factors such as an incomplete immunological response to vaccines, undiagnosed immunodeficiency disorders, and the relative immunological immaturity observed in preterm infants. Moreover, in January 2024, newborn screening for severe combined immunodeficiency was introduced, though its clinical impact is still under investigation in terms of the epidemiology and severity of immunodeficiency-related diseases [30,31,32].

Regarding the etiological agents, the most frequent were *S. pneumoniae*, *N. meningitidis*, and *S. agalactiae*, followed by *H. influenzae* and *S. pyogenes*, with their distribution varying by age group. The temporal trend of notifications showed significant variations over the analyzed period. From 2007 to 2019, there was a general decline in overall cases of pediatric IBDs. Then a sharp decrease was observed during the pandemic period, with a significant reduction in IBD cases due to *S. pneumoniae*, *H. influenzae*, and *N. meningitidis* in early 2020 coinciding with the introduction of COVID-19 containment measures. This trend was also observed in several countries and reported by other studies [19,33,34,35,36]. These epidemiological data may reflect the reduced interaction among individuals due to the COVID-19 restriction measures, which consequently led to a decrease in the transmission of common viral infections and a reduction in clinical evaluations in pediatric care [37]. The notification rate after the COVID-19 pandemic remains unclear. Our data demonstrated a significant rising trend in the number of cases across all age groups, consistent with estimates reported at the national level [36] and in other epidemiological studies [38]. The gradual easing of the restrictive measures imposed during the SARS-CoV-2 pandemic seems to have led to a subsequent resurgence of airborne pathogens. Conversely, other analyses have reported a relative decrease in the incidence of IBDs, but with a concomitant increase in severity and mortality in both pediatric and adult populations [39]. This uncertainty highlights the critical importance of ongoing surveillance to detect potential changes in the epidemiological landscape.

Notably, compared to other bacteria, the notification rates of *S. agalactiae* remained stable during the pandemic period. Unlike other bacteria transmitted via the respiratory route, *S. agalactiae* is primarily associated with maternal colonization during pregnancy, which is the main risk factor for the development of early neonatal infection [19,33,40].

Moreover, as observed in several European countries, our region also experienced a statistically significant upward trend in *S. pyogenes* infections across the pediatric population from 2019 to 2023. The ECDC has reported an alarming increase in invasive GAS infections since September 2022, particularly among children under ten years of age, likely associated with the increased circulation of respiratory viruses in the post-pandemic period, including seasonal influenza and respiratory syncytial virus (RSV) [15]. Coinfection with these viruses and GAS may have elevated the risk of invasive disease.

Overall, among the IPD cases caused by *S. pneumoniae* recorded between 2007 and 2023, 32.2% were due to PCV13 serotypes, 33.2% to PCV15 serotypes, and 38.7% to PCV20 serotypes.

By analyzing the overall distribution of serotypes, a significant decrease in the circulation of serotypes included in the PCV13 vaccine—available in the Veneto Region since 2014—can be observed. However, the consistent circulation of serotype 3, known for its resistance and immune evasion [41], remains notable.

Since 2014, the circulation of other serotypes, primarily included in the PCV20 vaccine, has emerged, with serotype 8 showing significant spread, similar to what has been observed in the elderly population [22]. From the same year, an increase in the circulation of non-vaccine serotypes has also been observed, while the proportion of non-typeable serotypes has remained stable, except during the pandemic period. According to the ECDC, at the European level, in recent years, the majority of IPD cases caused by *S. pneumoniae* appear to be associated with non-vaccine serotypes [10].

When stratifying by age and focusing on the 0–1 and 1–4 age groups, a near-total disappearance of serotypes covered by PCV13—except for serotype 3—has been noted since 2014, along with the sporadic emergence of some serotypes included in PCV15/PCV20.

Additionally, it is interesting that in the post-pandemic phase, serotype 19A has re-emerged in the 1–4 age group. This trend is particularly interesting due to the disappearance of vaccine-covered serotypes, which will need to be further assessed in the coming years following the end of the pandemic emergency and the introduction of broader-spectrum vaccines such as PCV15 in 2022.

The analysis of *N. meningitidis* serotypes responsible for pediatric IMDs revealed a notable increase in serotype B, accompanied by a decline in serotype C, which has not been detected since 2019. This shift in epidemiology is attributed to the introduction of vaccination against meningococcal C in 2005, leading to a significant reduction in IMD cases caused by serogroup C and a corresponding rise in cases caused by serogroup B [42,43]. IMDs caused by serotypes A, W, and Y were sporadic in our region.

Regarding *H. influenzae*, the majority of IBD cases were due to a not-typed serotype, whilst all typed cases (only 17.1% of the total) were associated with serotype b. In addition, for *H. influenzae*, there has been an increase in untyped cases since 2016, particularly in the 1–4 and 5–9 age groups, although the trends were not statistically significant in the Joinpoint analysis.

The latest report from the Italian Ministry of Health on pediatric vaccination coverage shows satisfactory percentages. In 2022, at 24 months of age, approximately 95.33%, 91.23%, 91.57%, 90.64%, and 93.96% of children in the Veneto Region were vaccinated against *H. influenzae* type b; meningococcus C, B, and ACYW; and pneumococcus, respectively. These coverage rates are generally higher than the national averages [44].

These high vaccination coverage rates could explain the overall decline in pediatric IBD cases as attributable to the effectiveness of vaccination strategies implemented over the past two decades.

They may suggest that the increases observed after the COVID-19 emergency are more likely reflective of both an increase in surveillance reporting and a return to social interactions.

To improve the accuracy of the regional notification rate trends for IBDs, it would be beneficial not only to compare these data with national-level data but also to make the specifications outlined in the reporting form mandatory, particularly when completed using digital tools. Although reporting IBD cases is mandatory, the accuracy of the information submitted to the surveillance system depends on the awareness of the reporting operator.

Indeed, in a recent evaluation of the Italian National Surveillance System for IBDs, Rosu et al. found that among the notifications received between 2016 and 2022 for *Haemophilus influenzae*, the completeness of data—especially regarding vaccination status, place of symptom onset, outcome, and serotype—was suboptimal, ranging between 39% and 87%. Additionally, timeliness, assessed as the time between symptom onset and first notification, varied substantially by region, ranging from 3 to 27 days on average [45]. Studies like these are essential for the continuous improvement of surveillance systems. An alternative approach could be to integrate notification data with other sources—for instance, hospital discharge records (HDRs). This method would not only help identify cases diagnosed with an IBD for which no infectious disease notification has been received but also serve as a strategy to assess the effectiveness of surveillance by quantifying metrics such as sensitivity and specificity. For example, Baldovin and colleagues observed improved accuracy by combining data from the mandatory notification system with HDRs and laboratory reports using the capture−recapture method to estimate IMD cases [43].

Our research has strengths and limitations. First, as reported in a previous study using records from the same surveillance system [22,26], only cases reported in the Veneto Region were included, which may restrict the applicability of the findings to other populations. However, our results generally mirror the Italian estimates from national surveillance [36]. Additionally, the dataset relies solely on information provided in the notification forms, as accessing complete medical histories was not feasible. Vaccination status and follow-up data, including information on sequelae and mortality, were often incomplete, as they may be unavailable to clinicians, and limited to notification date. These gaps limit our ability to fully evaluate the long-term outcomes of invasive bacterial diseases (IBDs) and the impact of vaccination.

Moreover, changes in surveillance practices and notification form over time and potential reporting biases may have influenced the results. Although the surveillance system is highly specific in identifying severe cases, its sensitivity is limited. The pandemic period, 2020–2021, further complicated the analysis, as the number of notifications, and likely the bacterial isolation, as well as the typed cases, dropped significantly due to shifts in healthcare priorities and reporting practices. As a result, surveillance data may also introduce quantitative biases, potentially underestimating both IBD cases and associated mortality in epidemiological assessments.

## 5. Conclusions

This study highlights the importance of the surveillance system in the Veneto Region as the primary source of data on the epidemiology of invasive bacterial diseases in the pediatric population. These data enable persistent and accurate surveillance of serotype distribution in the general population. This is crucial for assessing the impact of vaccination and monitoring serotype replacement with a view to introducing new vaccination strategies. In particular, monitoring serotype replacement can be strategic for evaluating the various epidemiological trends of future serotype circulation.

The results of this study show a decrease in cases of invasive bacterial diseases in the pre-COVID-19 period within the pediatric population, supporting the effectiveness of current vaccination policies. However, at the same time, the post-pandemic upward trend observed in 2022–2023 highlights the significant role of ongoing monitoring to inform future public health programs. Furthermore, the epidemiological data on *S. agalactiae* could play a role in future pandemics caused by airborne pathogens, serving as a control for non-respiratory pathogens to monitor any laboratory disruptions caused by the pandemic [33].

Further studies are needed to address current surveillance limitations, potentially improving the system by utilizing integrated approaches that rely both on clinical data, such as HDRs, and on laboratory monitoring to increase notification sensitivity.

These surveillance results confirm the importance of pediatric vaccination for protection against invasive infections and the necessity for improvements in timeliness and completeness of surveillance reports to obtain more accurate epidemiological estimates.

Evidence-based vaccination strategies and vaccine adherence promotion in pediatric age remain essential public health tools to protect not only children but also other vulnerable populations, especially the elderly, by achieving herd immunity [22,46].

## Figures and Tables

**Table 1:** Characteristics of pediatric invasive bacterial disease notifications in the Veneto Region from 2007 to 2023, stratified by age group.

Variable		Age (Years)															Total			*p*-Value *
		0			1–4			05-Set			10–14			15+			0–17			
		*n*	c %	r %	*n*	c %	r %	*n*	c %	r %	*n*	c %	r %	*n*	c %	r %	*n*	c %	r %	
Etiological agent	*Streptococcus pneumoniae*	77	38.7	26.4	119	65.4	40.8	70	76.9	24.0	15	51.7	5.1	11	32.4	3.8	292	54.6	100	<0.001
	*Neisseria meningitidis*	29	14.6	27.6	39	21.4	37.1	8	8.8	7.6	9	31.0	8.6	20	58.8	19.0	105	19.6	100	
	*Streptococcus agalactiae*	70	35.2	97.2	0	0.0	0.0	2	2.2	2.8	0	0.0	0.0	0	0.0	0.0	72	13.5	100	
	*Haemophilus influenzae*	17	8.5	41.5	13	7.1	31.7	7	7.7	17.1	3	10.3	7.3	1	2.9	2.4	41	7.7	100	
	*Streptococcus pyogenes*	6	3.0	24.0	11	6.0	44.0	4	4.4	16.0	2	6.9	8.0	2	5.9	8.0	25	4.7	100	
Sex	Female	86	43.2	38.7	78	42.9	35.1	35	38.5	15.8	9	31.0	4.1	14	41.2	6.3	222	41.5	100	0.729
	Male	113	56.8	36.1	104	57.1	33.2	56	61.5	17.9	20	69.0	6.4	20	58.8	6.4	313	58.5	100	
Notification season	Autumn	57	28.6	36.5	61	33.5	39.1	23	25.3	14.7	7	24.1	4.5	8	23.5	5.1	156	29.2	100	0.174
	Spring	47	23.6	33.8	46	25.3	33.1	31	34.1	22.3	8	27.6	5.8	7	20.6	5.0	139	26.0	100	
	Summer	34	17.1	54.0	14	7.7	22.2	6	6.6	9.5	3	10.3	4.8	6	17.6	9.5	63	11.8	100	
	Winter	61	30.7	34.5	61	33.5	34.5	31	34.1	17.5	11	37.9	6.2	13	38.2	7.3	177	33.1	100	
Associated	Meningitis	80	40.2	41.2	53	29.1	27.3	27	29.7	13.9	13	44.8	6.7	21	61.8	10.8	194	36.3	100	<0.001
diseases	Sepsis	138	69.3	45.1	90	49.5	29.4	52	57.1	17.0	11	37.9	3.6	15	44.1	4.9	306	57.2	100	<0.001
	Bacteremic pneumonia	10	5.0	12.3	44	24.2	54.3	23	25.3	28.4	1	3.4	1.2	3	8.8	3.7	81	15.1	100	<0.001
	Other	11	5.5	31.4	13	7.1	37.1	6	6.6	17.1	4	13.8	11.4	1	2.9	2.9	35	6.5	100	<0.001
Material	Blood	135	67.8	39.0	122	67.0	35.3	64	70.3	18.5	14	48.3	4.0	11	32.4	3.2	346	64.7	100	<0.001
	Liquor	74	37.2	43.5	44	24.2	25.9	21	23.1	12.4	10	34.5	5.9	21	61.8	12.4	170	31.8	100	0.046
	Other	1	0.5	14.3	3	1.6	42.9	3	3.3	42.9	0	0.0	0.0	0	0.0	0.0	7	1.3	100	0.38
Sequelae	All types	2	1.0	40.0	1	0.5	20.0	1	1.1	20.0	1	3.4	20.0	0	0.0	0.0	5	0.9	100	0.461
	Audiological	0	0.0	0.0	1	0.5	50.0	0	0.0	0.0	1	3.4	50.0	0	0.0	0.0	2	0.4	100	0.151
	Neurological	1	0.5	100	0	0.0	0.0	0	0.0	0.0	0	0.0	0.0	0	0.0	0.0	1	0.2	100	1
	Other	2	1.0	66.7	0	0.0	0.0	1	1.1	33.3	0	0.0	0.0	0	0.0	0.0	3	0.6	100	0.578
Deceased	Yes	6	9.4	25.0	10	18.2	41.7	3	12.5	12.5	3	20.0	12.5	2	15.4	8.3	24	14.0	100	0.603
	NA	135			127			67			14			21			364			
Total		199	100	37.2	182	100	34.0	91	100	17.0	29	100	5.4	34	100	6.4	535	100	100	

Legend: NA, not available; c %, column percentage; r %, row percentage. * *p*-Values refer to the statistical testing of differences between age groups in column percentages.

## Data Availability

The data that support the findings of this study are available on request from the corresponding author.

## Supplementary Materials

The following supporting information can be downloaded at: https://www.mdpi.com/article/10.3390/vaccines13030230/s1, Figure S1: Trend of serotype distribution for pediatric invasive bacterial disease caused by *Streptococcus pneumoniae* in the Veneto Region from 2007 to 2023 stratified by age: (A) 0 year, (B) 1–4 years, (C) 5–9 years, (D) 10–14 years, (E) ≥15 years; Figure S2: Trend of serotype distribution for pediatric invasive bacterial disease caused by *Neisseria meningitidis* in the Veneto Region from 2007 to 2023 stratified by age: (A) 0 year, (B) 1–4 years, (C) 5–9 years, (D) 10–14 years, (E) ≥15 years; Figure S3: Trend of serotype distribution for pediatric invasive bacterial disease caused by *Haemophilus influenzea* in the Veneto Region from 2007 to 2023 stratified by age: (A) 0 year, (B) 1–4 years, (C) 5–9 years, (D) 10–14 years, (E) ≥15 years.

## Appendix A

**Table A1:** Veneto Region vaccination strategies to contrast IBDs.

Microorganism	Vaccination Evolution in the Veneto Region
*N. meningitidis* B	The meningococcal B (MenB) vaccine is administered in a three-dose schedule: the first dose at 76 days after birth, the second at 151 days after birth, and the third at 13 months of age. The MenB vaccine was first introduced in the Veneto Region for newborns of the 2015 cohort. A significant change was implemented in August 2023, as the region now offers the MenB vaccine to all adolescents and young adults born between 1997 and 2009. This updated vaccination strategy aims to address the rising number of meningococcal B cases among young people, observed since the resumption of normal social interactions post-pandemic. The vaccine is actively offered, with invitations extended to newly turned 14-year-olds [47].
*N. meningitidis* ACWY	Vaccination against meningococcal serogroups A, C, W, and Y (MenACWY) is recommended at one year of age, with a booster dose provided for adolescents at 13 years of age. The MenACWY vaccine replaced the MenC vaccine in the Veneto Region starting in 2015. Vaccination against serogroup C was initially introduced in Veneto Region in 2005 and was offered to newborns at 13 months of age. This vaccination was later extended to 15-year-old adolescents and, starting in 2008, to 6-year-old children in response to an increase in meningococcal disease cases caused by this strain [48].
*S. pneumoniae*	The routine schedule for the conjugate pneumococcal vaccine (PCV) provides the first dose at two months of age, the second dose at four months, and the final dose at ten months of age. The PCV for newborns was introduced in the Veneto Region in 2006. Previously, the 23-valent polysaccharide pneumococcal vaccine (PPSV23) was available, but it is now used only in sequential combination. Initially, in 2002, the conjugate vaccine covered seven serotypes (PCV7): 4, 6B, 9V, 14, 18C, 19F, and 23F. In 2010, it was expanded to PCV10, which added serotypes 1, 5, and 7F, and subsequently to PCV13 in 2014, which further included serotypes 3, 6A, and 19A. More recently, a 15-valent vaccine, which additionally covers serotypes 22F and 33F, was authorized by AIFA (Agenzia Italiana del Farmaco) in 2022. A 20-valent vaccine, which also includes serotypes 8, 10A, 11A, 12F, and 15B, was approved by the EMA (European Medicines Agency) in 2022. Additionally, a 21-valent vaccine, incorporating a new subset of serotypes (3, 6Aa, 7F, 8, 9N, 10A, 11A, 12F, 15A, 15Cab, 16F, 17F, 19A, 20, 22F, 23A, 23B, 24F, 31, 33F, and 35B), was licensed by the FDA (U.S. Food and Drug Administration) in 2024, though it is not yet available in Europe [49].
*H. influenzae*	Regarding H. influenzae, the routine schedule of the Hib vaccine (as part of the hexavalent vaccine) has consisted of three doses co-administered with pneumococcal vaccine since 2002. The vaccine against H. influenzae type b was introduced in the Veneto Region in 1999 as part of the pediatric vaccination program. Initially, it was administered as a monovalent vaccine or in combination with other vaccines. The Hib vaccine is mandatory based on Italian law [50].
