# Supplementary material for: Surveillance of Pediatric Invasive Bacterial Diseases in the Veneto Region: Epidemiological Trends and Outcomes over 17 Years (2007–2023)
Source: Vaccines (Basel). 2025 Feb 24;13(3):230. doi: 10.3390/vaccines13030230 (PMC11945320; doi:10.3390/vaccines13030230)
Supplement: Supplementary file 1 [file vaccines-13-00230-s001.zip › vaccines-3468777-supplementary.pdf]

Supplementary materials

Figure S1. Trend of serotypes distribution for pediatric Invasive Bacterial Disease caused by *Streptococcus pneumoniae* in the Veneto Region from 2007 to 2023 stratified by age: (A) 0 year, (B) 1-4 years, (C) 5-9 years, (D) 10-14 years, (E) ≥15 years.

A

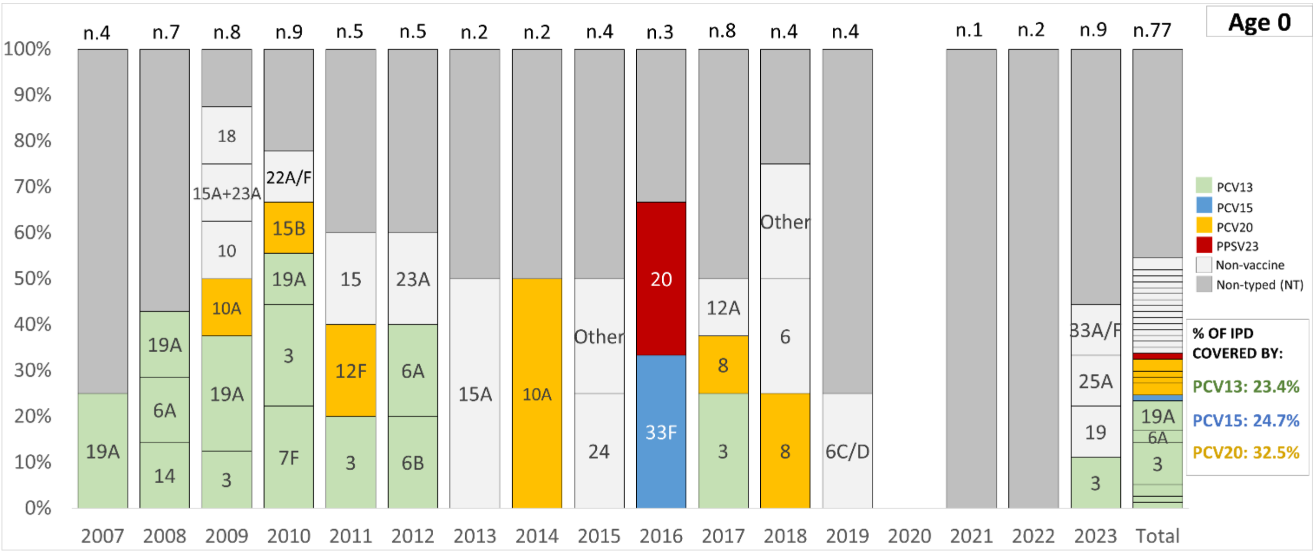

B

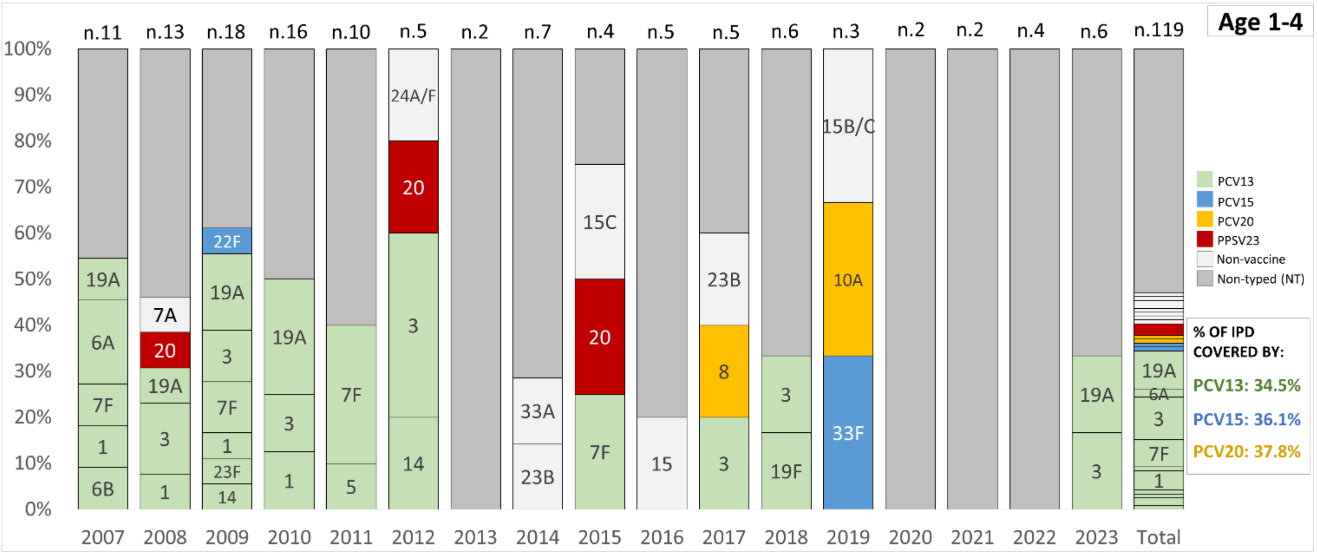

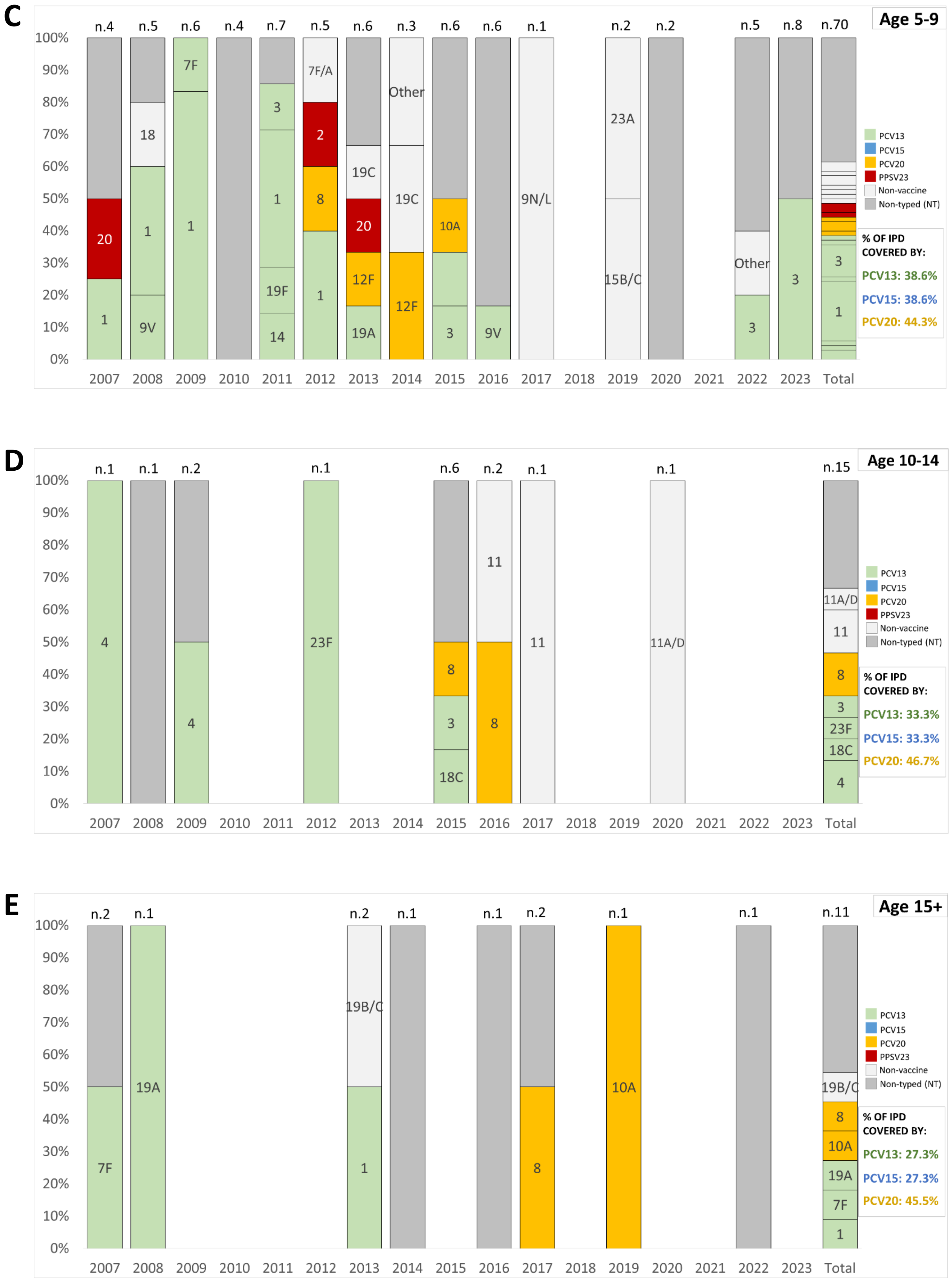
C
Age 5-9
n.4 n.5 n.6 n.4 n.7 n.5 n.6 n.3 n.6 n.6 n.1 n.2 n.2 n.5 n.8 n.70
100% 90% 80% 70% 60% 50% 40% 30% 20% 10% 0%
2007 2008 2009 2010 2011 2012 2013 2014 2015 2016 2017 2018 2019 2020 2021 2022 2023 Total
PCV13
PCV15
PCV20
PPSV23
Non-vaccine
Non-typed (NT)
% OF IPD COVERED BY:
PCV13: 38.6%
PCV15: 38.6%
PCV20: 44.3%
D
Age 10-14
n.1 n.1 n.2 n.1 n.6 n.2 n.1 n.1 n.15
100% 90% 80% 70% 60% 50% 40% 30% 20% 10% 0%
2007 2008 2009 2010 2011 2012 2013 2014 2015 2016 2017 2018 2019 2020 2021 2022 2023 Total
PCV13
PCV15
PCV20
PPSV23
Non-vaccine
Non-typed (NT)
% OF IPD COVERED BY:
PCV13: 33.3%
PCV15: 33.3%
PCV20: 46.7%
E
Age 15+
n.2 n.1 n.2 n.1 n.1 n.2 n.1 n.1 n.11
100% 90% 80% 70% 60% 50% 40% 30% 20% 10% 0%
2007 2008 2009 2010 2011 2012 2013 2014 2015 2016 2017 2018 2019 2020 2021 2022 2023 Total
PCV13
PCV15
PCV20
PPSV23
Non-vaccine
Non-typed (NT)
% OF IPD COVERED BY:
PCV13: 27.3%
PCV15: 27.3%
PCV20: 45.5%

Figure S2. Trend of serotypes distribution for pediatric Invasive Bacterial Disease caused by *Neisseria meningitidis* in the Veneto Region from 2007 to 2023 stratified by age: (A) 0 year, (B) 1-4 years, (C) 5-9 years, (D) 10-14 years, (E) ≥15 years.

A

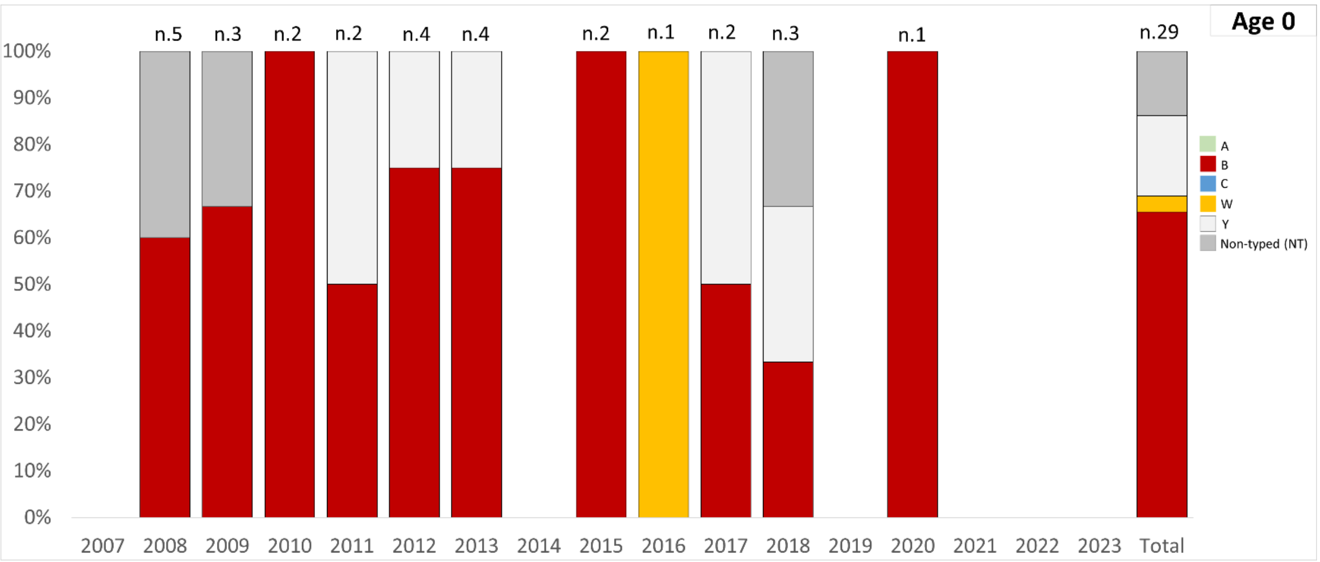

B

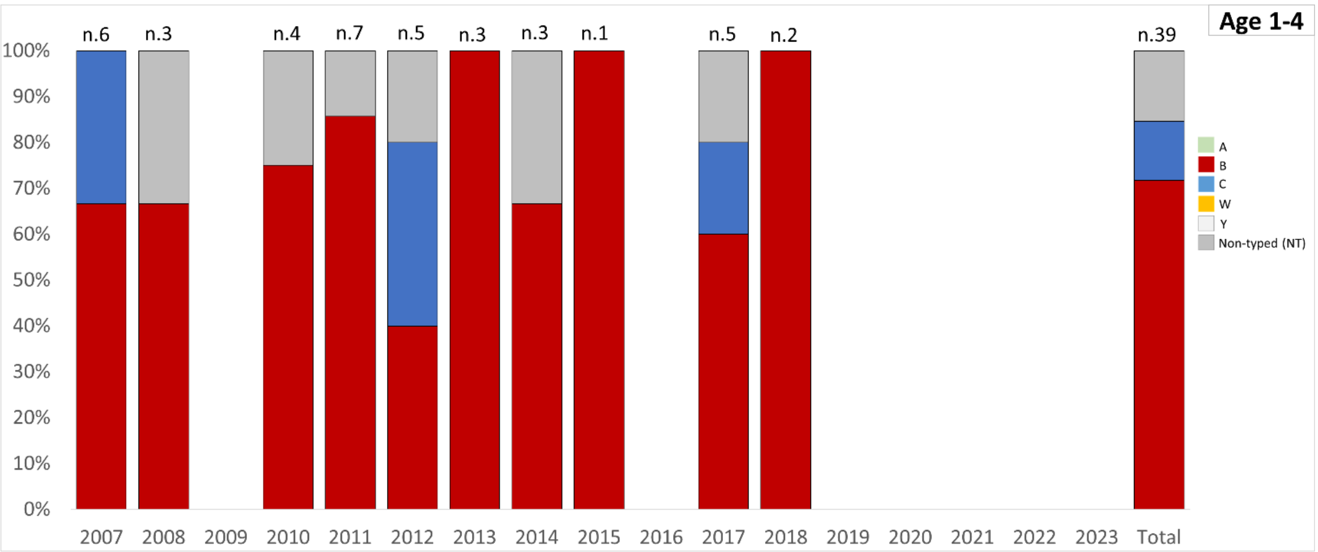

C

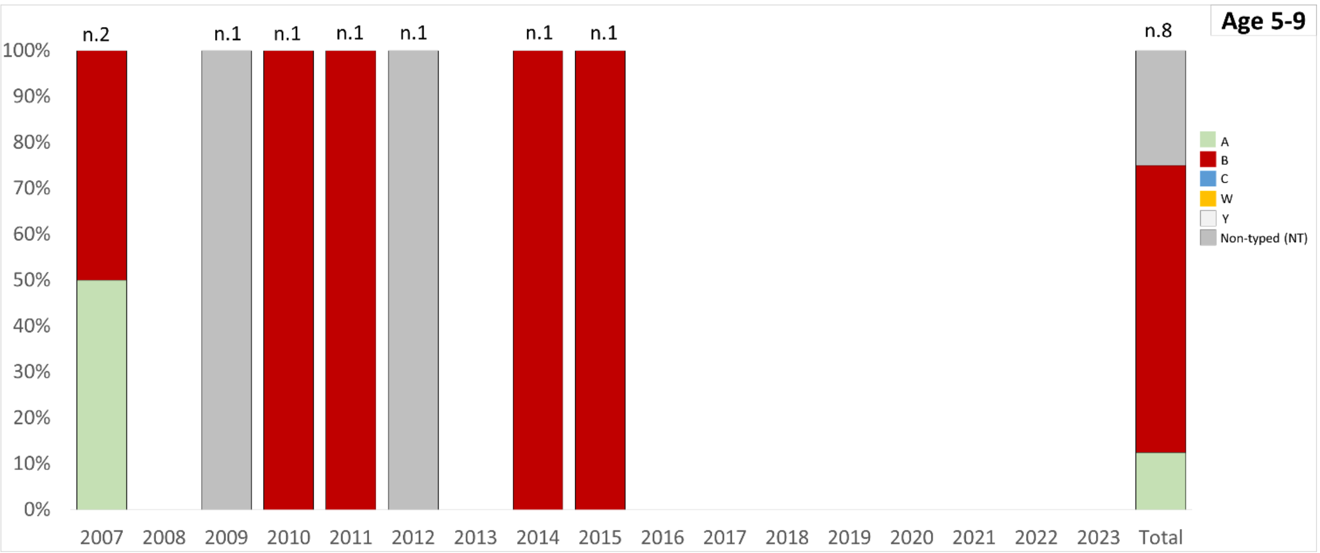
Age 5-9
n.2
n.1
n.1
n.1
n.1
n.1
n.1
n.8
100%
90%
80%
70%
60%
50%
40%
30%
20%
10%
0%
2007 2008 2009 2010 2011 2012 2013 2014 2015 2016 2017 2018 2019 2020 2021 2022 2023 Total
A
B
C
W
Y
Non-typed (NT)

D

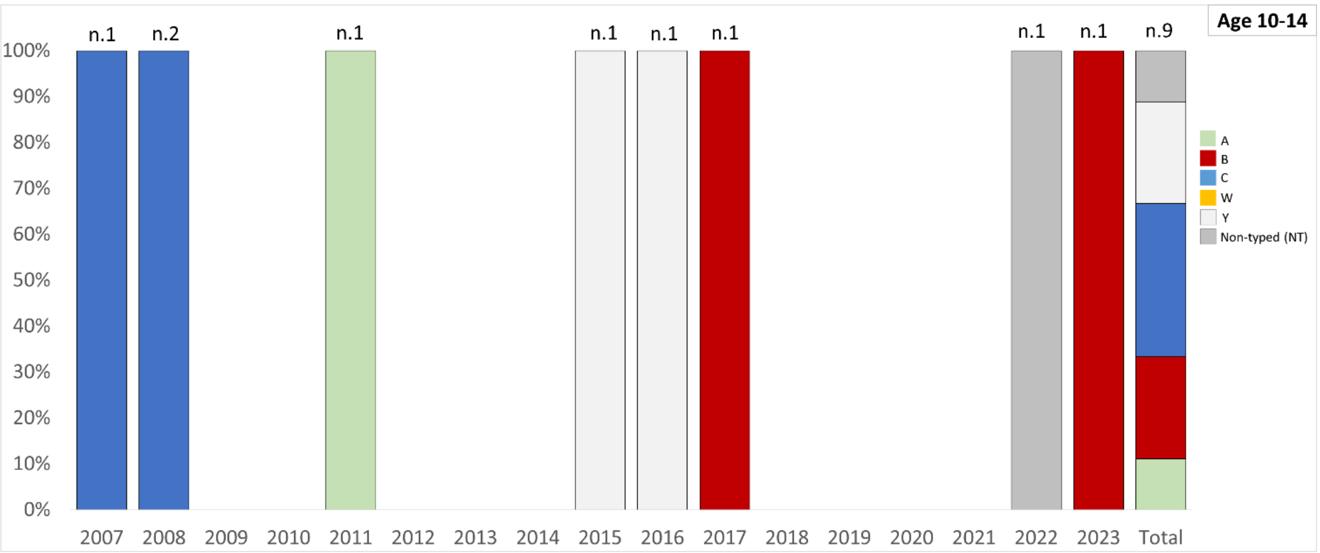
Age 10-14
n.1
n.2
n.1
n.1
n.1
n.1
n.1
n.1
n.9
100%
90%
80%
70%
60%
50%
40%
30%
20%
10%
0%
2007 2008 2009 2010 2011 2012 2013 2014 2015 2016 2017 2018 2019 2020 2021 2022 2023 Total
A
B
C
W
Y
Non-typed (NT)

E

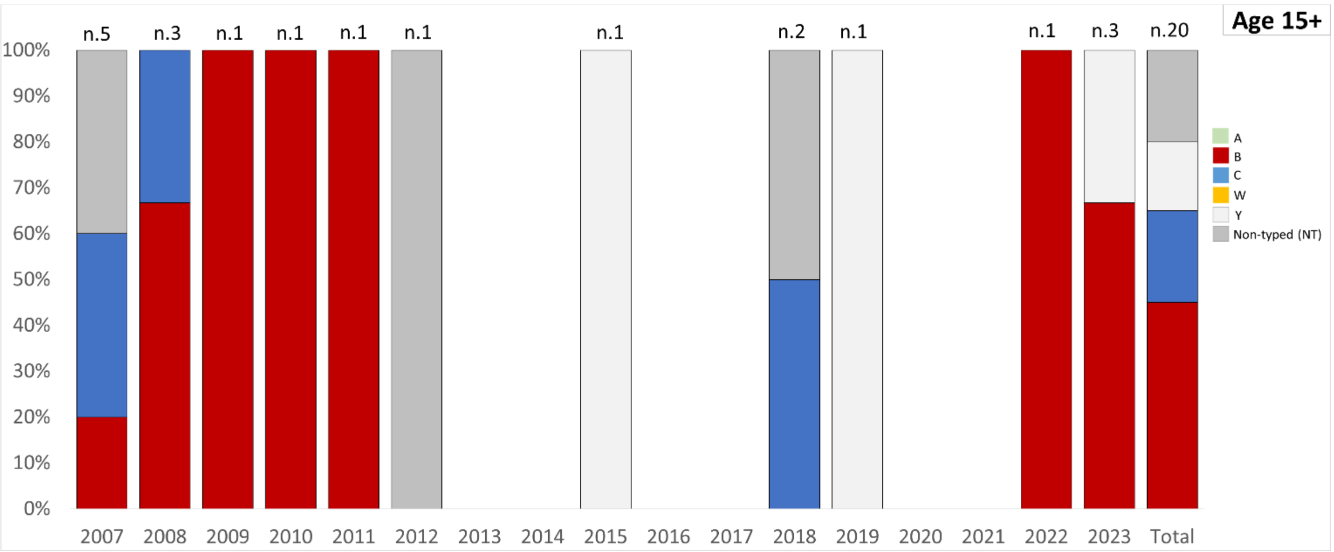
Age 15+
n.5
n.3
n.1
n.1
n.1
n.1
n.1
n.2
n.1
n.1
n.3
n.20
100%
90%
80%
70%
60%
50%
40%
30%
20%
10%
0%
2007 2008 2009 2010 2011 2012 2013 2014 2015 2016 2017 2018 2019 2020 2021 2022 2023 Total
A
B
C
W
Y
Non-typed (NT)

Figure S3. Trend of serotypes distribution for pediatric Invasive Bacterial Disease caused by *Haemophilus influenzea* in the Veneto Region from 2007 to 2023 stratified by age: (A) 0 year, (B) 1-4 years, (C) 5-9 years, (D) 10-14 years, (E) ≥15 years.

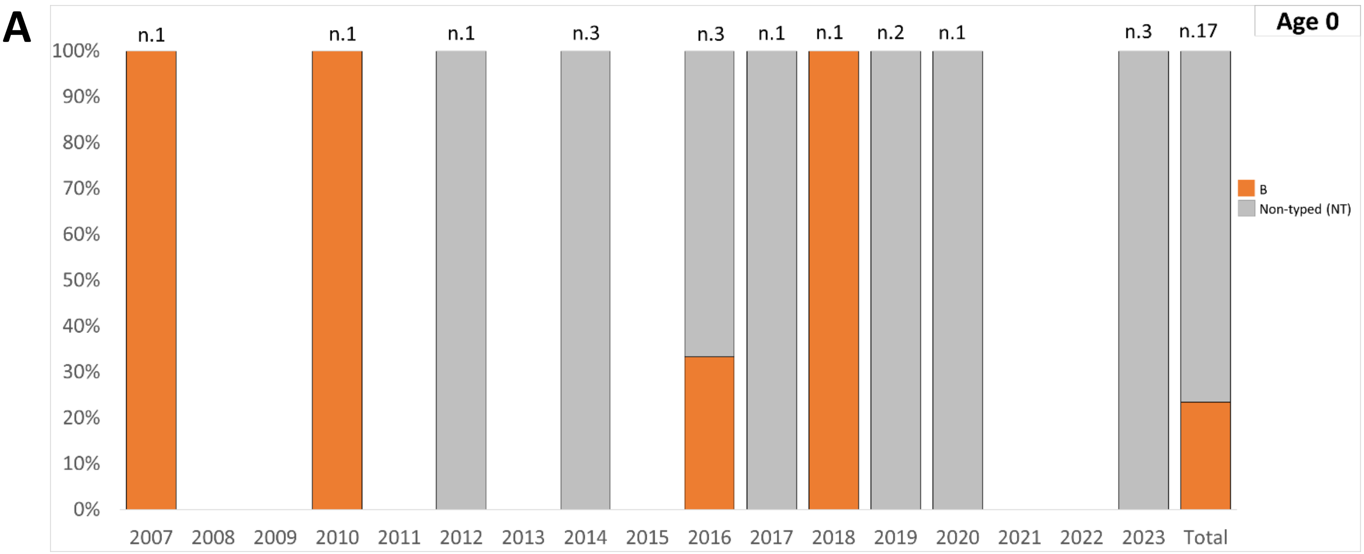

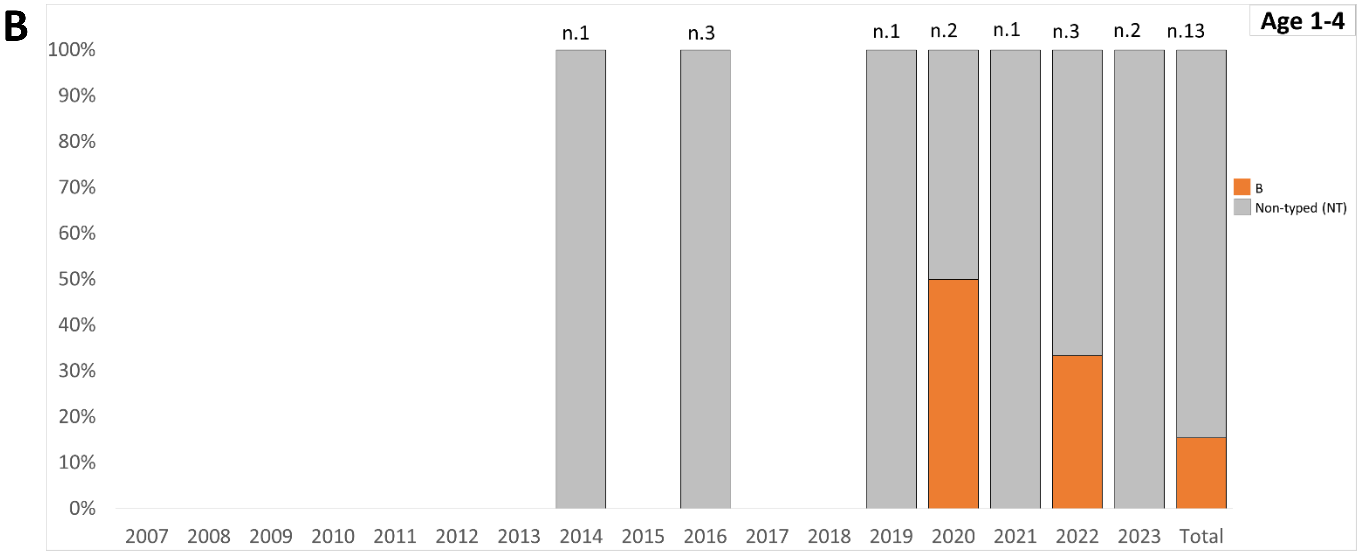

C

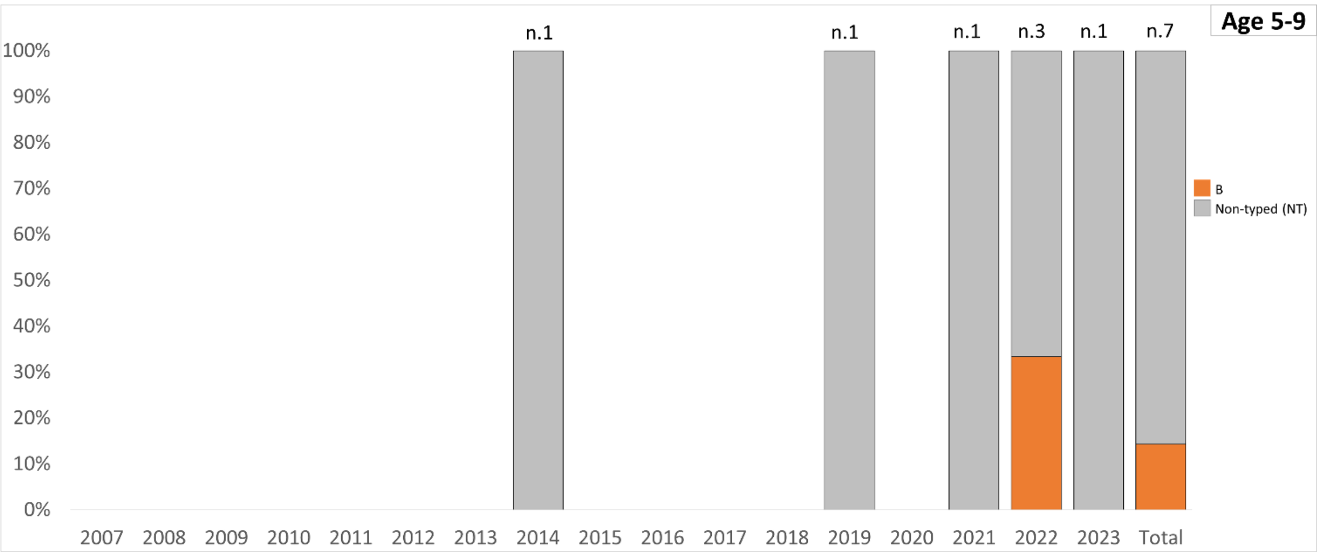
Age 5-9
n.1
n.1
n.1
n.3
n.1
n.7
100%
90%
80%
70%
60%
50%
40%
30%
20%
10%
0%
2007 2008 2009 2010 2011 2012 2013 2014 2015 2016 2017 2018 2019 2020 2021 2022 2023 Total
B
Non-typed (NT)

D

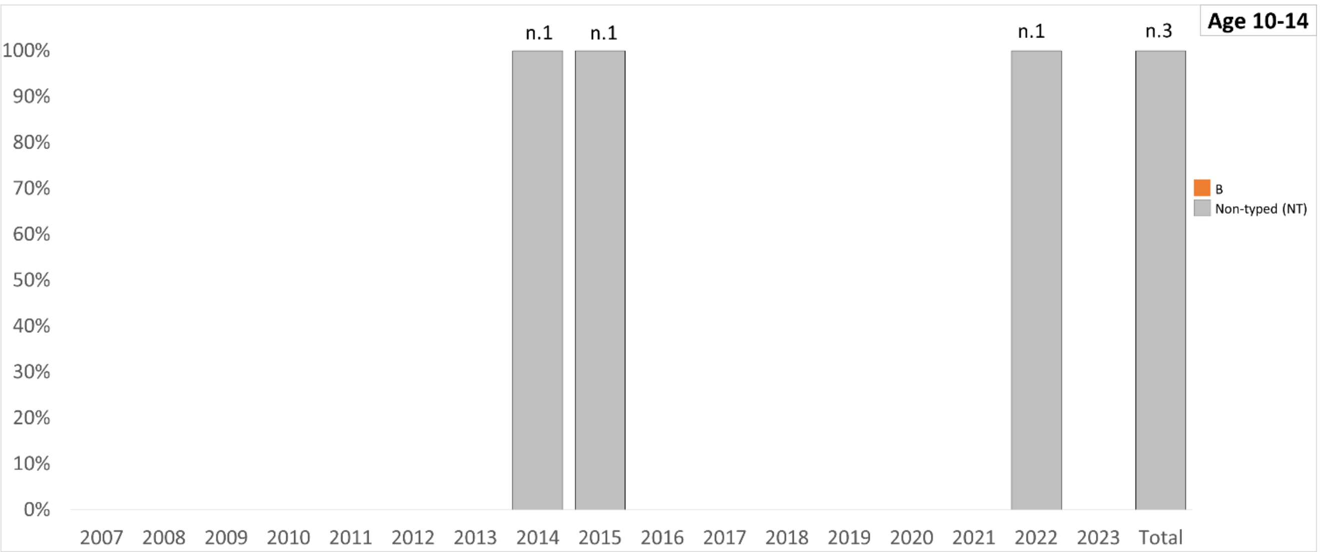
Age 10-14
n.1
n.1
n.1
n.3
100%
90%
80%
70%
60%
50%
40%
30%
20%
10%
0%
2007 2008 2009 2010 2011 2012 2013 2014 2015 2016 2017 2018 2019 2020 2021 2022 2023 Total
B
Non-typed (NT)

E

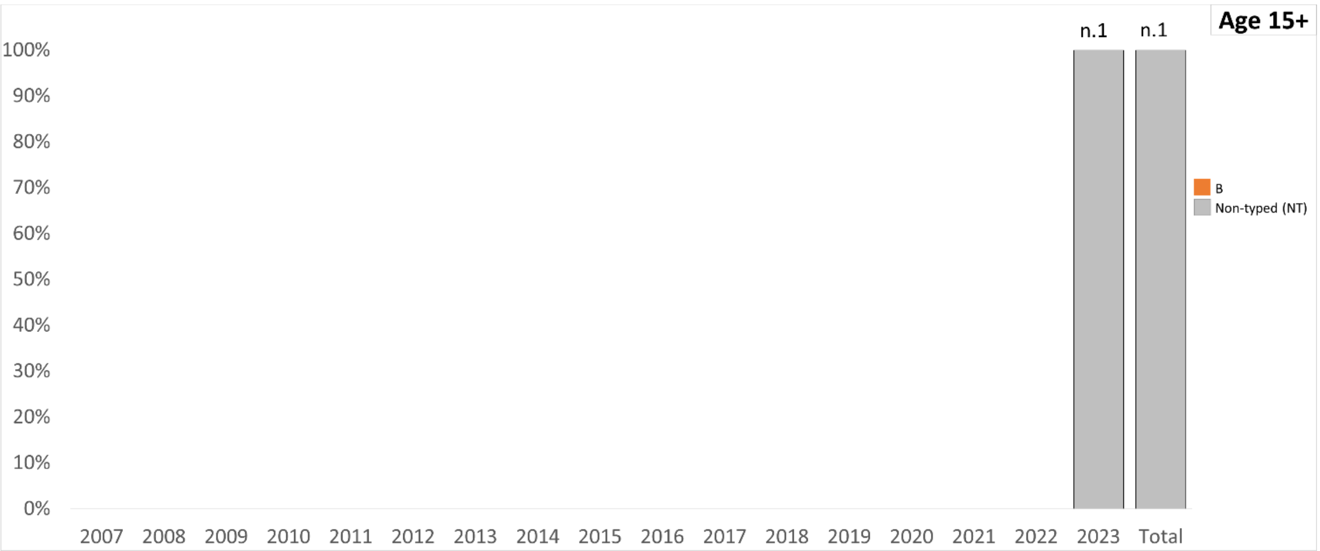
Age 15+
n.1
n.1
100%
90%
80%
70%
60%
50%
40%
30%
20%
10%
0%
2007 2008 2009 2010 2011 2012 2013 2014 2015 2016 2017 2018 2019 2020 2021 2022 2023 Total
B
Non-typed (NT)
